# Physical Activity as a Predictor of Cognitive Decline in an Elderly Essential Tremor Cohort: A Prospective, Longitudinal Study

**DOI:** 10.3389/fneur.2021.658527

**Published:** 2021-05-20

**Authors:** Keith H. Radler, Silvia Chapman, Maria Anna Zdrodowska, Hollie N. Dowd, Xinhua Liu, Edward D. Huey, Stephanie Cosentino, Elan D. Louis

**Affiliations:** ^1^Department of Neurology, University of Texas (UT) Southwestern Medical Center, Dallas, TX, United States; ^2^Department of Neurology, College of Physicians and Surgeons, Columbia University, New York, NY, United States; ^3^Taub Institute for Research on Alzheimer's Disease and the Aging Brain, College of Physicians and Surgeons, Columbia University, New York, NY, United States; ^4^Division of Movement Disorders, Department of Neurology, Yale School of Medicine, Yale University, New Haven, CT, United States; ^5^Department of Biostatistics, College of Physicians and Surgeons, Columbia University, New York, NY, United States; ^6^Department of Psychiatry, College of Physicians and Surgeons, Columbia University, New York, NY, United States

**Keywords:** essential tremor, cognitive aging, physical activity, cerebellar diseases, movement disorders

## Abstract

**Background:** Essential tremor (ET), one of the most common neurological diseases, is associated with cognitive impairment. Surprisingly, predictors of cognitive decline in ET remain largely unidentified, as longitudinal studies are rare. In the general population, however, lower physical activity has been linked to cognitive decline.

**Objectives:** To determine whether baseline physical activity level is a predictor of cognitive decline in ET.

**Methods:** One hundred and twenty-seven ET cases (78.1 ± 9.5 years, range = 55–95), enrolled in a prospective, longitudinal study of cognition. At baseline, each completed the Physical Activity Scale for the Elderly (PASE), a validated, self-rated assessment of physical activity. Cases underwent an extensive battery of motor-free neuropsychological testing at baseline, 1.5 years, and 3 years, which incorporated assessments of cognitive subdomains. Generalized estimating equations (GEEs) were used to assess the predictive utility of baseline physical activity for cognitive change.

**Results:** Mean follow-up was 2.9 ± 0.4 years (range = 1.3–3.5). In cross-sectional analyses using baseline data, lower physical activity was associated with lower overall cognitive function as well as lower cognitive scores in numerous cognitive domains (memory, language, executive function, visuospatial function and attention, all *p* < 0.05). In adjusted GEE models, lower baseline physical activity level significantly predicted overall cognitive decline over time (*p*=0.047), and declines in the subdomains of memory (*p* = 0.001) and executive function (*p* = 0.03).

**Conclusions:** We identified reduced physical activity as a predictor of greater cognitive decline in ET. The identification of risk factors often assists clinicians in determining which patients are at higher risk of cognitive decline over time. Interventional studies, to determine whether increasing physical activity could modify the risk of developing cognitive decline in ET, may be warranted.

## Introduction

Essential tremor (ET) is one of the most common neurological diseases, affecting approximately seven million patients in the US (1). While the primary motor feature of ET is a kinetic tremor that can interfere with upper limb function (2), ET cases may also experience a mild form of gait ataxia that has been associated with loss of confidence in balance and a greater propensity for near-falls and actual falls (3–6). ET is also associated with cognitive impairment. Indeed, ET cases display poorer cognitive performance and an increased prevalence of mild cognitive impairment (MCI) compared to age-matched controls (7–9). ET cases are also at increased risk of developing incident dementia (10, 11).

With few exceptions, data that describe cognitive impairment in ET are cross-sectional rather than longitudinal (12) and there are few longitudinal cohorts other than that of the current study. Besides older age, additional variables have yet to be identified as predictors of cognitive decline in ET (12). In one cross-sectional study of participants with ET, an association was identified between lower levels of physical activity and poorer performance on the Montreal Cognitive Assessment (MOCA), a common measure of global cognition (13). Similarly, lower physical activity has been identified as a longitudinal predictor of conversion to MCI (14) and dementia (15–18) in the general population. Research in Parkinson's disease (PD), a related movement disorder, shows that lower physical activity is associated with greater cognitive impairment (19). Along these lines, a variety of data suggest the neuroprotective capacity of physical activity (20). Randomized controlled trials have shown that increased physical activity in MCI cases in the general population led to improved cognition, suggesting that the neuroprotective benefits of physical activity extend to those who are most at risk of developing dementia (21). Preliminary data in ET and extensive data in the general population support the potential predictive capacity of physical activity on cognitive change.

The Physical Activity Scale for the Elderly (PASE, described in greater detail below) is a frequently used geriatric self-report scale designed to assess the level of physical activity of older adults in epidemiological studies (22, 23). Here we examine PASE scores and their relationship to cognitive change in a cohort of elderly ET cases, enrolled in a prospective, longitudinal cohort study of cognition in ET. We hypothesize that low levels of physical activity will predict cognitive decline in this cohort.

This study is poised to specify the potential effect of physical activity in ET by examining longitudinal data across multiple domains of cognition. In doing so, we hope to identify what could be a modifiable risk factor for particular types of cognitive impairment in ET, a finding which would have significant implications for clinicians and patients. The identification of modifiable predictors could shift the clinical dialogue surrounding cognitive impairment in ET toward lifestyle changes and ultimately, improved outcomes.

## Methods

### Study Design

The COGNET study (Clinical-Pathological Study of Cognitive Impairment in Essential Tremor, NINDS R01NS086736) is an ongoing, prospective, longitudinal study of cognitive function in ET. The study began in July 2014 as an effort to identify the profiles, neuroanatomic bases, prevalence and course of cognitive impairment in ET cases. As described previously (24), cases who met the following eligibility criteria were recruited: (1) diagnosis of ET, (2) minimum age of 55 years, (3) no brain surgery for the treatment of ET, (4) willingness to participate in testing and enroll as a brain donor. Recruitment took place through advertisements on a study website and other websites (International Essential Tremor Foundation). Demographic and clinical data including age, gender, ethnicity, and education were collected at baseline. All portions of the study were approved by the Yale University, Columbia University, and University of Texas Southwestern Internal Review Boards, and all cases granted signed, informed consent upon enrollment.

### Study Evaluation

Eligible cases underwent an extensive motor-free neuropsychological battery during in-person research visits at three regularly interspersed intervals: at baseline (T1), 18 months after baseline (T2), and 36 months after baseline (T3). A neuropsychologist (S.C.) designed the cognitive test battery to have minimal to no reliance on motor functioning. The 4-h battery targets several cognitive domains including attention, executive function, visuospatial function, language, and memory. A detailed description of the battery and the neuropsychological tests has been described previously (24). Other variables of interest were also collected, including age of tremor onset and number of prescription medications, among others. At each of the three visits, cases were asked to designate a close relative or friend to act as an informant. Research staff interviewed the designated informant regarding the case's cognition and everyday function and assigned each case a Clinical Dementia Rating Score (0 = no dementia, 0.5 = questionable dementia, 1 = mild dementia, 2 = moderate dementia, and 3 = severe dementia) (CDR) reviewed and confirmed by a geriatric psychiatrist (E.D.H.) (25).

Together, the cognitive test scores, informant interview and CDR supplied information for clinical diagnoses assigned by a clinical neuropsychologist (S.C.) and geriatric psychiatrist (E.D.H.) during diagnostic case conferences at each of the three intervals. Cases were assigned one of three primary cognitive diagnoses: normal cognition (ET-NC), mild cognitive impairment (ET-MCI), or dementia (ET-D) (24). Cases with neuropsychological test impairment in at least two domains and a CDR ≥1 were considered clinically demented whereas those with a CDR score = 0 and impairment on one test or less were considered normal. MCI was defined as a CDR of 0.5 and impairment (z-score ≤ −1.5) on 2 MCI-designated tests. Seven cases with baseline diagnosis of ET-D were excluded from this analysis.

During the baseline visit, baseline physical activity was measured using the PASE. The PASE is designed to measure the level of physical activity in the last 7 days in older adults, and consists of 10 items divided into three different categories: leisure time activity, household activity, and work-related activity. The first six items measure how frequently and for how many hours per day the individual engages in physical activity of varying intensity for leisure. The following three items use yes or no questions to assess engagement in household physical activity. The final item queries whether respondents are involved in work for pay or as a volunteer and the physical exertion involved in such work. Responses are entered into a scoring algorithm, which delivers an overall physical activity score with higher scores indicating greater physical activity. Scores can range from 0 to 400 or more (26). This 10 item questionnaire is a commonly used and validated self-report scale for the measure of physical activity (22, 23).

Self-reported depression was measured using the Geriatric Depression Scale (GDS), a 30-item self-report measure of depressive symptoms in older adults. Scores range from 0 to 30 with higher scores indicating more depressive symptoms (27). Cases completed the GDS during each of the three study visit intervals.

Self-reported anxiety was measured using the General Anxiety Disorder Seven-Item (GAD-7) scale (28). The GAD-7 consists of seven questions related to anxiety symptoms. Scores for each question range from 0 to 3, with greater scores indicating greater anxiety symptom severity. Total GAD-7 scores range from 0 to 21, with higher scores indicating more severe general anxiety. Cases completed the GAD-7 at the second and third study visits.

As an additional measure of comorbidity, we used a modified version of the Cumulative Illness Rating Scale (29). Cases describe illnesses and ailments related to 14 different organ systems. Based on their response, a score from 0 to 3 is assigned for each of the 14 organ systems, with higher scores indicating greater severity of illness. Scores can theoretically range from 0 to 42, with higher scores indicating greater comorbidity. Modified versions of the CIRS have been used extensively as an indicator of comorbidity in geriatric populations (30, 31). In order to maximize use of CIRS data, which was collected at T2 and T3, an average CIRS score from two visits was used as an overall indicator of cumulative illness.

Trained research staff filmed a neurological diagnostic examination at each of the three intervals. A senior movement disorder specialist (E.D.L.) confirmed diagnoses of ET by evaluating the neurological examination according to the Washington Heights-Inwood Genetic Study of ET diagnostic criteria (32), which requires moderate or greater amplitude kinetic tremor during three or more tests or head tremor in the absence of PD, dystonia, or other causes. These criteria have been shown to be reliable (33) and valid (34). Total tremor score was assigned by a movement disorders neurologist (E.D.L.) following examination of the videotaped neurological examination. The total tremor score (range 0–36, higher scores indicate more severe tremor) was calculated based on ratings (0–3) for kinetic or postural tremor on 12 movement tasks and assigned at each of the three intervals (24). Balance impairment was measured using a tandem gait exercise. Cases were asked to walk 10 steps in a straight path with the heel of the leading food touching the toe of the following foot. The number of tandem gait missteps, or steps off of the straight line, was the reported outcome.

### Cognitive Variables

To standardize values for cognitive outcome variables, raw scores from neuropsychological testing were transformed into z-scores based on the current sample. The neuropsychological raw scores of those who had a diagnosis of ET-NC at baseline served as the basis for the development of standardized Z-scores. The means and standard deviations from this sample of cognitively normal individuals with ET were used to assign z scores in cognition for all cases at each of the three intervals. This within-sample method of developing z-scores offers advantages over the use of published normative data for each test as the within-sample z-scores are based on the same sample, eliminating any differences in cognitive outcomes that may be related to the specific norms used. Second, within sample norms based on cognitively normal ET cases minimize any disadvantage on cognitive testing which may possibly be related to tremor (although any effects are expected to be minimal on the selected tests). The categories of cognition for which z scores were developed include overall cognition and five subdomains: memory, executive function, language, attention, and visuospatial function. This method of domain aggregation has been described previously in the context of this cohort (24).

### Statistical Analysis

There were 18 ET cases who did not complete the PASE questionnaire at baseline, leaving 127 ET cases. Variables were described by mean and standard deviation for continuous measures and number and percentage for categorical measures (Table 1). We used Spearman correlation coefficients to detect bivariate associations between baseline quantitative variables (Table 2).

**Table 1:** Demographic and clinical characteristics, including PASE and cognitive scores, of 127 ET cases at three time intervals.

	**T1**	**T2**	**T3**
Age (years)	78.1 ± 9.5	79.6 ± 9.6	80.8 ± 9.4
Male	48 (37.8)	-	-
Education (years)	15.8 ± 2.6	-	-
Number of prescription medications	5.4 ± 3.7	5.8 ± 3.7	5.9 ± 3.2
CIRS^a^	-	6.3 ± 3.4	7.9 ± 3.9
Cognitive diagnosis			
Normal cognition	108 (85.0)	101 (79.5)	92 (78.6)
MCI	19 (15.0)	22 (17.3)	15 (11.8)
Dementia	-	4 (3.2)	10 (7.9)
CDR			
0	102 (80.3)	88 (69.3)	79 (62.2)
0.5	25 (19.7)	36 (28.3)	29 (22.8)
1	-	3(2.4)	5 (3.9)
2	-	-	3 (2.4)
Age of tremor onset (years)	38.9 ± 21.3	-	-
Total tremor score	19.9 ± 4.8	20.9 ± 5.2	19.4 ± 5.1
Tandem gait missteps	4.4 ± 4.0	5.2 ± 3.9	5.9 ± 4.1
GDS score	6.1 ± 5.0	6.7 ± 4.9	7.3 ±5.2
GAD-7 score^a^	-	2.9 ± 3.75	3.0 ± 3.3
PASE score	97.8 ± 72.7	84.7 ± 61.8	83.3 ± 58.4
Cognitive Z scores			
Overall	−0.16 ± 0.63	−0.09 ± 0.77	−0.17 ± 0.92
Memory	−0.17 ± 0.90	−0.17 ± 1.03	−0.04 ± 1.21
Executive function	−0.13 ± 0.65	−0.18 ± 0.89	−0.24 ± 0.95
Attention	−0.11 ± 0.84	−0.08 ± 0.99	−0.23 ± 0.95
Language	−0.25 ± 1.19	0.10 ± 1.14	−0.10 ± 1.50
Visuospatial	−0.13 ± 0.74	−0.11 ± 0.90	−0.22 ± 0.95

*Values are reported as mean ± standard deviation or as number (percent)*.

*When data were missing, total n was <127*.

a *Data not collected at T1*.

*CDR, clinical dementia rating; CIRS, cumulative illness rating scale; GAD-7, general anxiety disorder seven-item; GDS, geriatric depression scale; MCI, mild cognitive impairment; PASE, physical activity scale for the elderly*.

**Table 2:** Relationship between baseline variables and baseline PASE.

	**Correlation coefficient with baseline PASE (*r*)**	***p-*value**
Age	**−0.43**	***p*** **<** **0.001**
Male	0.11	0.20
Education	0.08	0.35
Number of prescription medications	–**0.22**	***p*** **=** **0.016**
CIRS^a^	–**0.33**	**0.004**
Cognitive diagnosis	–**0.20**	***p*** **=** **0.025**
Normal		
MCI		
CDR	−0.12	*p* = 0.14
0		
0.5		
Age of tremor onset	−0.06	0.50
Total tremor score	−0.12	0.41
Tandem gait missteps	–**0.36**	***p*** **<** **0.001**
GDS score	–**0.33**	***p*** **<** **0.001**
GAD-7 score^b^	0.017	0.85
**Cognitive Z scores**		
Overall	**0.43**	***p*** **<** **0.001**
Memory	**0.22**	***p*** **=** **0.015**
Executive function	**0.39**	***p*** **<** **0.001**
Attention	**0.33**	***p*** **<** **0.001**
Language	**0.30**	***p*** **=** **0.001**
Visuospatial	**0.36**	***p*** **<** **0.001**

*Values are Spearman correlation coefficients for bivariate associations (r)*.

a *Average of T2 and T3 values is baseline measure*.

b *T2 is baseline measure*.

*CDR, clinical dementia rating; CIRS, cumulative illness rating scale; GAD-7, general anxiety disorder seven-item; GDS, geriatric depression scale; MCI, mild cognitive impairment; PASE, physical activity scale for the elderly*.

*Bolded values represent significant correlations*.

The generalized estimating equation (GEE) approach, which uses all available data and is robust to misspecification of correlation structure in repeated measures, was used to estimate and test hypotheses on the model parameters of interest (Tables 3, 4). This method offers the advantage of incorporating all available data from all time points.

**Table 3:** Estimated parameters of linear models with interaction with repeated measures for cognitive change over time.

**Overall cognition**	***B* (se)**	***p*-value**
**Unadjusted model**		
Time from baseline (years)	−0.007 (0.0182)	0.695
Baseline PASE	0.070 (0.0121)	< .001
Time by baseline PASE interaction	0.007 (0.0038)	0.051
**Adjusted model**		
Time from baseline (years)	−0.012 (0.0171)	0.471
Baseline PASE	0.025 (0.0119)	0.035
Time by baseline PASE interaction	**0.007 (0.0036)**	**0.047**
Baseline age	−0.038 (0.0050)	<0.001
Male vs. female	−0.036 (0.1011)	0.724
Baseline education	0.012 (0.0216)	0.582
Baseline number of medications	−0.163 (0.0544)	0.003
**Memory**	***B*** **(se)**	***p*****-value**
**Unadjusted model**		
Time from baseline (years)	0.045 (0.0243)	0.067
Baseline PASE	0.052 (0.0205)	0.011
Time by baseline PASE interaction	**0.015 (0.0051)**	**0.003**
**Adjusted model**		
Time from baseline (years)	0.037 (0.0228)	0.107
Baseline PASE	0.011 (0.0212)	0.614
Time by baseline PASE interaction	**0.015 (0.0048)**	**0.001**
Baseline age	−0.036 (0.0086)	<0.001
Male vs. female	−0.328 (0.1539)	0.033
Baseline education	0.027 (0.0295)	0.361
Baseline number of medications	−0.185 (0.0764)	0.015
**Executive function**	***B*** **(se)**	***p*****-value**
**Unadjusted model**		
Time from baseline (years)	−0.045 (0.0204)	0.029
Baseline PASE	0.066 (0.0137)	<0.001
Time by baseline PASE interaction	**0.008 (0.0038)**	**0.034**
**Adjusted model**		
Time from baseline (years)	−0.050 (0.0196)	0.011
Baseline PASE	0.019 (0.0138)	0.181
Time by baseline PASE interaction	**0.008 (0.0036)**	**0.030**
Baseline age	−0.034 (0.0055)	<0.001
Male vs. female	−0.060 (0.1132)	0.596
Baseline education	0.029 (0.0234)	0.209
Baseline number of medications	−0.255 (0.0668)	<0.001

*Square root transformation applied to baseline PASE score and number of medications*.

*Bolded values represent significant time by baseline PASE interactions*.

*GDS, geriatric depression scale; PASE, physical activity scale for the elderly; se, standard error*.

**Table 4:** Estimated parameters in the linear models without interaction with repeated measures for cognitive change over time.

	**Language**		**Attention**		**Visuospatial function**	
	***B* (se)**	***p*-value**	***B* (se)**	***p*-value**	***B* (se)**	***p*-value**
**Unadjusted model**						
Time (years)	0.043 (0.0335)	0.194	−0.042 (0.0226)	0.064	−0.033 (0.0231)	0.147
Baseline PASE	0.079 (0.0229)	0.001	0.079 (0.0168)	<0.001	0.076 (0.0157)	<0.001
**Adjusted model**						
Time (years)	0.050 (0.0333)	0.133	−0.038 (0.0203)	0.064	−0.032 (0.0221)	0.153
Baseline PASE	0.032 (0.0215)	0.132	0.027 (0.0194)	0.163	**0.031 (0.0155)**	**0.045**
Baseline age	−0.040 (0.0087)	<0.001	−0.046 (0.0072)	<0.001	−0.036 (0.0064)	<0.001
Male vs. female	0.237 (0.1742)	0.174	−0.073 (0.1368)	0.593	0.013 (0.1191)	0.916
Education	−0.002 (0.0418)	0.965	0.002 (0.0252)	0.932	−0.006 (0.0248)	0.816
Number of medications	−0.073 (0.1186)	0.538	−0.157 (0.0643)	0.015	−0.171 (0.0609)	0.005

*Square root transformation applied to PASE score and number of medications*.

*When the time by PASE interaction term was not significant, the model excludes that term, and it is not represented in one of the rows*.

*Bolded values represent significant baseline PASE interaction*.

*PASE, physical activity scale for the elderly; se, standard error*.

We fit several models to the data. The initial model examined the effect of baseline PASE score on change in each cognitive domain score. In adjusted models we added baseline demographic covariates including age, sex, education, as well as number of prescription medications as a measure of morbidity. We examined the impact of including total tremor score and age of tremor onset in the models, however, since these variables were not related to cognitive outcomes, they were not included.

We extended the models by including terms for the physical activity by time interaction. The coefficients of the interaction terms indicated whether baseline physical activity predicted rate of change in the outcome over time. The final model excluded non-significant interaction terms to prevent masking the main effects of time and/or PASE score.

We also performed a sensitivity analysis that included additional covariates: baseline number of tandem gait missteps as a measure of balance, baseline GDS as an indicator of depressive symptoms, and CIRS as an additional indicator of comorbidity. GAD-7 score was considered but was left out, as it was not related to baseline PASE or cognitive outcomes. We ran these models only in domains which yielded a significant interaction term in our adjusted models. As the number of covariates was large in these additional models, therefore potentially leading to a loss of statistical power, and many of these covariates were co-linear (e.g., age and tandem gait missteps), therefore making it difficult to disentangle independent effects, the aim of these analyses was to assess whether the size of beta value (i.e., our effect estimate) changed significantly.

Statistical tests were two-sided and significance level was set at 0.05. All statistical analyses were conducted using SAS version 9.4 (SAS Institute, Cary, NC).

## Results

### Baseline Relationships

One hundred and twenty-seven ET cases aged 55–95 years (mean = 78.1 ± 9.5 years) were included in this analysis. See Table 1 for a full description of the demographic and clinical features of the 127 ET cases at each time point.

Baseline PASE scores ranged from 0 to 366.4, with a median score of 85 and a mean of 97.8. Lower baseline PASE score (lower level of physical activity) was associated with higher baseline GDS score (*r* = −0.33, *p* < 0.001), more prescription medications at baseline (*r* = −0.22, *p* = 0.016), higher CIRS score (*r* = −0.33, *p* = 0.004), greater number of tandem gait missteps (*r* = −0.36, *p* < 0.001), and older age at baseline (*r* = −0.43, *p* < 0.001).

**Figure 1:**
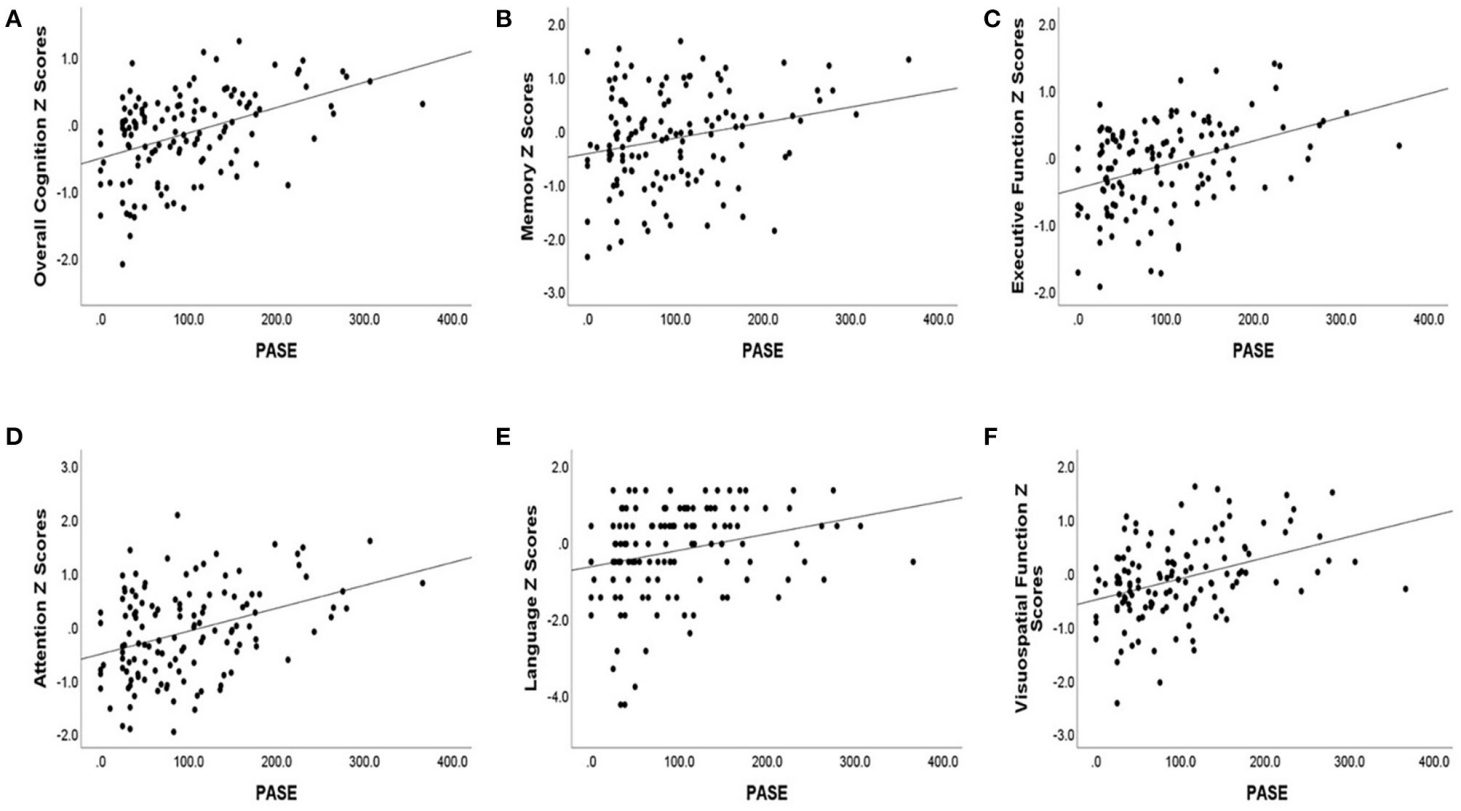
Baseline cognitive Z scores [**(A)** overall cognition, **(B)** memory, **(C)** executive function, **(D)** attention, **(E)** language, **(F)** visuospatial function] are plotted against baseline PASE score. Baseline cognitive scores are plotted against overall cognition and cognitive domain scores.

Lower baseline PASE score was also correlated with lower baseline cognitive test scores. Baseline PASE score was positively correlated with baseline z scores of overall cognition (*r* = 0.43, *p* < 0.001), as well as with the subdomains of attention, visuospatial function, executive function, (*r* = 0.33, 0.36, 0.39; *p* < 0.001), language (*r* = 0.30, *p* = 0.001), and memory (*r* = 0.22, *p* = 0.015). See Table 2 for the correlation coefficients describing the relationship between baseline cognitive measures and baseline PASE scores. See Figures 1A–F for a series of scatter plots depicting the relationship between baseline PASE and significant correlates.

As expected, older baseline age was significantly correlated with lower cognitive z scores in overall cognition (*r* = −0.63, *p* < 0.001), memory (*r* = −0.44, *p* < 0.001), executive function (*r* = −0.54, *p* < 0.001), attention (*r* = −0.49, *p* < 0.001), language (*r* = −0.36, *p* < 0.001), and visuospatial function (*r* = −0.56, *p* < 0.001).

### Linear Models With Repeated Measures

We examined time trends in cognitive outcomes and the impact of baseline PASE scores on cognitive change, with and without adjusting for covariates.

There were 127 ET cases with a total of 294 observations of overall cognition, memory, executive function, and attention over three visits (127 + 93 + 74 = 294). Excluding cases with missing baseline scores, there were 293 observations of language (126 + 93 + 74) from 126 cases and 288 observations of visuospatial function (126 + 92 + 70) from 126 cases.

Significant time by baseline PASE score interactions were observed in overall cognition, memory, and executive function (see bolded values in Table 3). These significant interaction terms suggest that lower physical activity is related to the time trend in overall cognition, as well as to the time trend in the subdomains of memory and executive function, with control for baseline demographics and number of medications (Table 3). In other words, baseline PASE score predicted change in overall cognition, memory, and executive function. One unit decrease in the square root of baseline PASE score was associated with a covariates-adjusted decrease in the time trend (Z-score per year) of 0.007 (95% CI: 0.000–0.014) for overall cognition, 0.015 (95% CI: 0.006–0.025) for the memory subdomain, and 0.008 (95% CI: 0.001–0.015) for the executive function subdomain. In summary, low baseline PASE score predicted decline in overall cognition, memory, and executive function.

We also performed a sensitivity analysis in which we included additional covariates: baseline number of tandem gait missteps as a measure of balance, baseline GDS as an indicator of depressive symptoms, and CIRS as an additional indicator of comorbidity. With the inclusion of additional balance, psychiatric, and comorbidity covariates, the size of the beta value did not change for the time by baseline PASE score interaction observed in overall cognition (*B* = 0.006, whereas B in our main analysis had been 0.007), for the time by baseline PASE score interaction observed in the memory domain (*B* = 0.017, whereas in our main analysis had been 0.015), or for the time by baseline PASE score interaction observed in executive function (*B* = 0.005, whereas *B* in our main analysis had been 0.008).

There was no significant baseline PASE score by time interaction on the language, attention, and visuospatial function subdomains, suggesting that baseline physical activity levels did not significantly affect the rate of change in these subdomains. Table 4 presents the models for these domains without the non-significant interaction term. In adjusted models, attention, and visuospatial scores declined over time with z score changes of −0.038 (95% CI: −0.077 to 0.002) and −0.032 (95% CI: −0.075 to 0.012) per year, respectively.

## Discussion

The goal of this study was to determine the prognostic utility of physical activity level for longitudinal cognitive outcomes. We show that baseline PASE score, a validated measure of physical activity in older adults, significantly impacts time trends in cognition and is predictive of cognitive change in ET. We showed that low physical activity was predictive of global cognitive decline in ET cases as well as decline in the subdomains of memory and executive function.

These findings contribute an important layer of understanding to a disorder that has already been associated with other indicators of poor health and outcomes in older adults such as balance and gait impairments (35), and poor functional activity (2, 36). Given the physical impairment and involuntary movements that are prominent in ET, it is likely that ET cases are at greater risk of significant reductions in physical activity. Indeed, the mean PASE score in this ET cohort (97.8) was lower than that of a cohort of older adults in the general population (155) (37) suggesting that ET cases may indeed engage in less physical activity. Further research is required to better define the relationship between tremor and engagement in physical activity, however the data presented here show that reduced physical activity predicts poorer cognitive outcomes in the ET population. Data such as these contribute to a more comprehensive and integrated understanding of one of the most common movement disorders (38).

The study of cognitive impairment in ET is a nascent field and longitudinal data describing the course of cognitive change are scarce (12). We know that ET cases exhibit a higher burden of cognitive impairment when compared to control populations (7–11) and that the rate of cognitive decline appears to be accelerated in ET cases (39). However, until this point, the only baseline feature identified as predictive of cognitive decline in ET was older age (10). Predictors for cognitive decline have been identified in the general population and in PD, yet they only partially overlap one another (12), indicating that predictors of cognitive decline in movement disorders, in general, including ET, cannot be assumed to be the same as those identified in the general populations. Hence, it is important to study ET populations to identify these predictors.

The identification of a modifiable risk factor for cognitive decline in ET is of relevance for clinicians and for the development of meaningful interventions. Physical activity is an adjustable variable that can be targeted and modified by lifestyle changes. A review of randomized controlled trials found that interventions involving increased physical activity in MCI cases resulted in improved cognition (21). Data presented here support the further study and critical testing of such interventions in ET cases. The identification of predictive variables for cognitive decline in ET may also help provide further guidance to researchers who aim to understand the unique features and neuroanatomic bases for poor cognition in this movement disorder.

A limitation to this study is the absence of a control group. Thus, it is beyond the scope of this study to draw any definitive conclusions regarding the unique impact that ET may have on physical activity or that the results presented here are unique to this population. However, we are able to conclude that in the context of ET, physical activity is a significant predictor of cognitive decline.

Given the absence of a control group, it can be useful to compare the results published here to those developed from other studies of physical activity in the general population. Several meta-analytic studies of the general population suggest that low physical activity is associated with cognitive decline (40, 41). Based on the findings we present here, this conclusion appears to extend to the ET population. In one randomized clinical trial of healthy adults, exercise was shown to have a positive impact on cognitive function. In particular, exercise was shown to have the greatest protective benefit on executive function in older individuals (42). Deficits in executive function are particularly pronounced in ET (43). The elevated protective benefit of physical activity on executive function may be even more consequential in the setting of ET as compared to settings where executive function is not the primary cognitive deficit. Indeed, in our study, physical activity significantly predicted rate of decline in executive function (*B* = 0.008, *p* = 0.030). A future direction for research might include a comparative study of the impact of physical activity on cognition in movement disorders and normal populations.

This study should be interpreted within the context of certain limitations. As described above, the absence of a control group poses a limitation to the conclusions we are able to draw from these results. As a result, we are only able to describe conclusions in the context of ET and cannot suggest that our findings are a direct result of living with ET. We are unable to compare with certainty the findings here to those in the general population. The data were obtained from a single sample of ET cases and cognitive scores were standardized within sample. As these volunteer cases self-referred, it is possible that they do not reflect the broader ET population. Physical activity was assessed with a self-report measure (PASE) rather than an objective measure. In some studies, PASE scores correlate with objective measures of energy expenditure; nonetheless this is not the case with all studies (44). Despite the self-report nature of the PASE, it is a standard and commonly-used measure employed in a wide range of research contexts (23, 26, 45). Some participants in the study were very old; this may be interpreted as a limitation as the very old are limited in their ability to engage in physical activity. Still, the range of PASE scores among the very old in our cohort was extensive; at baseline those 90 years of age or older had a range in PASE scores of 0–213.2. The mean baseline PASE score for the entire cohort was 97.8. Several of those aged 90 and above had a baseline PASE score higher than this mean. These findings suggest that physical activity in the very old is variable and that it is not safe to assume that the very old engage in little to no physical activity. This study is unable to make any definitive conclusions regarding the relationship between tremor and physical activity. Further research is required to determine whether the gait and balance issues associated with ET might contribute to reduced physical activity in this population. We are, however, able to conclude that in the *context* of ET, physical activity is predictive of cognitive change.

The study also had several strengths. First, inclusion of longitudinal data allowed us to assess the predictive capacity of physical activity for cognitive decline, rather than simply identifying a cross-sectional correlation. Given the scarcity of data describing longitudinal cognitive changes in ET cases (12), the inclusion of long term cognitive data is a considerable strength. The use of GEE analyses exploited the presence of longitudinal data as this method incorporates all available data at all time points. The outcome variables of cognition were ascertained from a comprehensive neuropsychological assessment of a community-based cohort of ET cases. Another positive aspect of the study includes the careful assignment of ET diagnoses by a movement disorders specialist to ensure an analysis of pure ET cases only. The assignment of cognitive diagnoses by neuropsychologists also is a strength, as the assignment of ET-NC diagnoses was critical for establishing the cognitive norms from which cognitive z scores were derived.

In conclusion, we present data describing the predictive capacity of physical activity level on longitudinal cognitive change in ET cases. In doing so, we support a growing foundation of research that describes the increased burden and unique features of cognitive impairment in movement disorders cases. Physical activity is an important health metric in general, and of particular relevance to the health and outcomes of those with ET. We provide further support for the importance of monitoring this modifiable risk factor clinically. Interventional studies, to determine whether increasing physical activity could modify the risk of developing cognitive decline in ET, may be warranted.

## Data Availability Statement

The raw data supporting the conclusions of this article will be made available by the authors, without undue reservation.

## Ethics Statement

The studies involving human participants were reviewed and approved by UT Southwestern IRB. The patients/participants provided their written informed consent to participate in this study.

## Author Contributions

KR: data curation, writing—original draft, writing—review, and editing. SC and XL: formal analysis, writing—review, and editing. MZ and HD: data curation, writing—review, and editing. EH: conceptualization, writing—review, and editing. SC and EL: conceptualization, writing—review, editing, and funding acquisition. All authors contributed to the article and approved the submitted version.

## Conflict of Interest

The authors declare that the research was conducted in the absence of any commercial or financial relationships that could be construed as a potential conflict of interest.
